# Does glenoid inclination affect the anterior stability of reverse total shoulder arthroplasty? A biomechanical study

**DOI:** 10.1007/s00590-024-03898-7

**Published:** 2024-04-09

**Authors:** Marc-Frederic Pastor, Dennis Nebel, Lennart Mathis Becker, Christof Hurschler, Alba Aurora Karrer, Tomas Smith

**Affiliations:** 1 Department of Orthopaedic Surgery, Städtisches Klinikum Braunschweig gGmbH, Holwedestraße 16, 38118 Braunschweig, Germany; 2 Department of Orthopaedic Surgery, DIAKOVERE Annastift, Hannover Medical School, Anna-von-Borries-Str. 1-7, 30625 Hannover, Germany; 3 Laboratory for Biomechanics and Biomaterials, Department of Orthopaedic Surgery, DIAKOVERE Annastift, Hannover Medical School, Anna-von-Borries-Str. 1-7, 30625 Hannover, Germany

**Keywords:** Reverse total shoulder arthroplasty, Instability, Inclination, Lateralisation

## Abstract

**Purpose:**

The anterior stability of reverse total shoulder arthroplasty is affected by multiple factors. However, the effect of glenosphere inclination on stability has rarely been investigated, which is what this study aims to look into.

**Methods:**

Reverse shoulder arthroplasty was performed on 15 cadaveric human shoulders. The anterior dislocation forces and range of motion in internal rotation in the glenohumeral joint (primary measured parameters) were tested in a shoulder simulator in different arm positions and implant configurations, as well as with a custom-made 10° inferiorly inclined glenosphere. The inclination and retroversion of the baseplate as well as the distance between the glenoid and coracoid tip in two planes (secondary measured parameters) were evaluated on CT scans.

**Results:**

In biomechanical testing, the custom-made inclined glenosphere showed no significant influence on anterior stability other than glenoid lateralisation over all arm positions as well as the neck-shaft angle in two arm positions. The 6 mm lateralised glenosphere reduced internal rotation at 30° and 60° of glenohumeral abduction. In 30° of glenohumeral abduction, joint stability was increased using the 155° epiphysis compared with the 145° epiphysis. The mean inclination was 16.1°. The inclination was positively, and the distance between the glenoid and coracoid tip in the anterior-to-posterior direction was negatively correlated with anterior dislocation forces.

**Conclusions:**

The custom-made inferiorly inclined glenosphere did not influence anterior stability, but baseplate inclination itself had a significant effect on stability.

## Introduction

Grammont-style reverse shoulder arthroplasty was developed to treat patients with end-stage rotator cuff deficiency with pseudoparalysis [1]. Since the inception of reverse total shoulder arthroplasty (RTSA), function has improved for every pre-operative diagnosis [2], the indications for the use of RTSA have broadened, and the number of patients treated with RTSA has steadily increased to 52% of all shoulder arthroplasties performed [3].

Nonetheless, some complications of RTSA remain, including instability, component loosening, impingement, periprosthetic fractures, and infections. Instability is observed in 2–31% after RTSA [4–7] and is associated with both patient- and implant-related factors, such as humeral cup depth, glenosphere size, soft tissue condition, compressive force of the deltoid muscle, and arm position [8–12]. The current literature indicates that glenoid inclination also influences RTSA stability. Two clinical studies by Randelli et al. and Tashjian et al. [13, 14] showed that inferior inclination of the glenoid was associated with a reduced risk of dislocation, and a greater superior baseplate inclination resulted in an increased risk for RTSA dislocation. However, another retrospective study did not find that prosthetic glenoid inclination or change in glenoid inclination significantly influenced RTSA stability [15]. An advantage of inferior tilt is that it minimises the shear force and maximises the compressive force and force distribution at the implant–bone interface [16]. However, it could also lead to impingement and increased scapular notching. Patel et al. [17] observed in a computer model that an inferior tilt of − 10° was associated with increased scapular neck impingement in external rotation at the side and adduction, increasing the risk of scapular notching and impingement-related instability. Scapular notching is another complication of RTSA [7] and is associated with inferior outcomes, a limited range of motion, and higher rates of complications [17–19]. Further, the lateralisation of the glenoid is associated with increased external rotation [20], but the influence of the lateralisation on internal rotation remains unclear.

To our knowledge, the effect of inferior tilt of the glenoid component of the RTSA has not been investigated in biomechanical studies. The aim of this study was to test the effect of custom-made 10° inferiorly inclined glenospheres in different arm positions on RTSA anterior stability. The hypothesis was that inferior inclination of the glenosphere leads to superior anterior stability. Furthermore, the correlation of anatomic landmarks, baseplate inclination and retroversion, implant position, and configuration of the RTSA were investigated, as well as the influence of glenoid lateralisation on internal rotation of the RTSA.

## Materials and methods

### Ethics approval

The Ethics Commission of the Hannover Medical School (No. MHH 8859_BO_K_2020) granted local ethics approval for this study.

### Sample size estimation

To determine the minimum number of specimens required to detect differences in stability between different implant configurations and joint positions, a power analysis was performed using G*Power 3.1 (G*Power, Düsseldorf, Germany) [21, 22]. Based on the results of two previous studies [12, 23] and assuming a power of 0.80 and an *α* of 0.05, the analysis showed that at least 12 specimens would be required.

### Specimen preparation and mounting

This study used 15 freshly frozen human shoulder specimens (Science Care Inc., Phoenix, AZ, USA). The median age of the 10 male and 5 female donors was 67.6 ± 11.3 years, and the body mass index was 24.3 ± 2.0 kg/m^2^.

Prior to preparation and testing, the specimens, stored at − 20 °C, were thawed at room temperature for 24 h. The lower part of the scapula was exposed by careful resection of soft tissue. The scapula was then embedded using a custom-made box and cold curing casting resin (Rencast FC 52/53 Isocyanate, FC 53 Polyol, Filler DT 982, Huntsman Corp., The Woodlands, TX, USA). In order to attain neutral rotation of the humerus, a Kirschner wire was drilled parallel to the axis of the forearm, while the elbow was held at 90° of flexion. The humerus was then cut approximately 20 cm distal to the centre of the humeral head and potted in a brass cylinder using a casting resin.

The scapular block was attached to a mounting plate by the use of three threaded rods with 10° of forward tilt to correspond to its physiological orientation to the thorax. This plate was mounted on the tower of the shoulder simulator, with the medial scapula margin oriented vertically in the coronal plane. A custom-made adapter on the flange of the robot was used to attach the potted cylinder to the humeral shaft of the shoulder simulator.

### Shoulder simulator

**Fig. 1:**
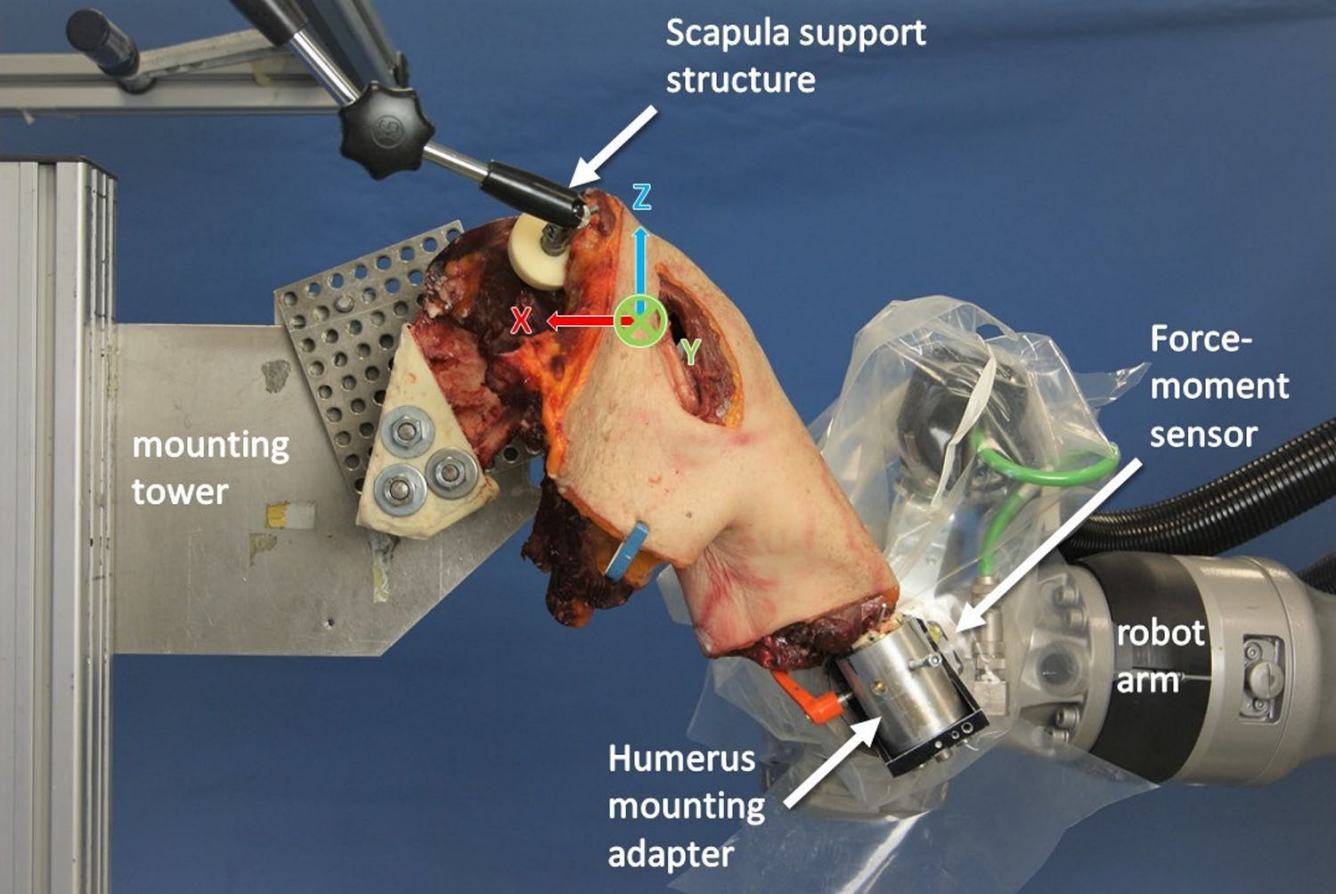
The shoulder simulator and the setup with a mounted shoulder specimen in 30° GH abduction and neutral rotation from anterior view with the coordinate system previously defined in the text. The humerus is attached to the force-moment sensor via a mounting adapter, which in turn is mounted to the end effector of the robot. The scapula is firmly attached to the mounting tower and additionally stabilised by an anterior support structure

The shoulder simulator (Fig. 1) consisted of an industrial robot (KR16-2, KUKA AG, Augsburg, Germany) equipped with a 6-component force-moment sensor (Delta, ATI Industrial Automation, Apex, NC, USA) and a mounting tower. While the humerus was attached to the flange of the robot arm, allowing motion of the glenohumeral joint by force and moment control, the scapula was rigidly attached to the mounting tower. The robot allows motion control with a repeatability of 0.04 mm and joint loading measurement with a resolution of less than 0.25 N and 7.5 N mm. Two coordinate systems were defined: A global coordinate system for robot control, and a specimen-specific coordinate system at the geometric centre of the humeral head. Regarding the global coordinate system, the *x*-axis was directed medially parallel to the previously defined scapular plane, the *y*-axis was defined as being perpendicular to the scapular plane pointing posteriorly, and the *z*-axis was defined orthogonal to the *x*- and *y*-axes and therefore pointing superiorly. A specimen-specific humeral coordinate system was used to describe the motion of the humerus with respect to the scapula. Before testing, the origin of this coordinate system, which simultaneously represents the geometric centre, had to be determined. For this purpose, passive elevation, flexion–extension, and rotation of the joint centred in the glenoid were performed using the force-moment-controlled robot while recording all positional data. Using an algorithm based on the least-squares method, the point which moved the least, the geometric centre of the humeral head, was calculated. The specimen-specific humeral coordinate system was oriented codirectionally to the global coordinate system after the glenohumeral joint was centred with the arm hanging under its own weight at 30° glenohumeral abduction (GH abduction) and neutral rotation.

### Surgical procedure

As described previously [12, 24], a deltopectoral approach was used to implant the reverse shoulder prosthesis (Delta Xtend; DePuy Synthes, Raynham, MA, USA). Before preparing the humerus, the supraspinatus and subscapularis tendons were resected, and tenotomy of the long head of the biceps was performed. Resection of the humeral head was conducted with an inclination angle of 155° and 10° retroversion. To achieve this, the cutting guide was aligned with a Kirschner wire in a 10° retroverted position of the humeral implant driver, which was placed in line with the forearm axis. The humeral shaft was then reamed to fit an appropriately sized press-fit stem into the humerus. To prepare the glenoid and obtain a clear view of the entire glenoid fossa, circumferential resection of the labrum and release of the remaining joint capsule were performed. By drilling the hole for the central peg of the metaglene near the centre of the inferior glenoid circle so that the border of the metaglene followed the inferior edge of the glenoid, the risk of scapular notching was minimised. The metaglene was then fixed to the glenoid using four 4.5-mm screws. Eccentric glenospheres with a diameter of 42 mm and varying degrees of lateralisation (0 mm and 6 mm) and inclination (0° and − 10°) were implanted depending on the test cycle. After surgery, the deltoid muscle, infraspinatus, teres minor, and conjoint tendons were intact, whereas the supraspinatus, long head of the biceps, and subscapularis tendon were resected, and the capsule was incised around the glenoid rim.

### Testing protocol

In our protocol, eight implant configurations with three varying parameters were investigated in four arm positions, resulting in 32 conditions for each specimen.

**Fig. 2:**
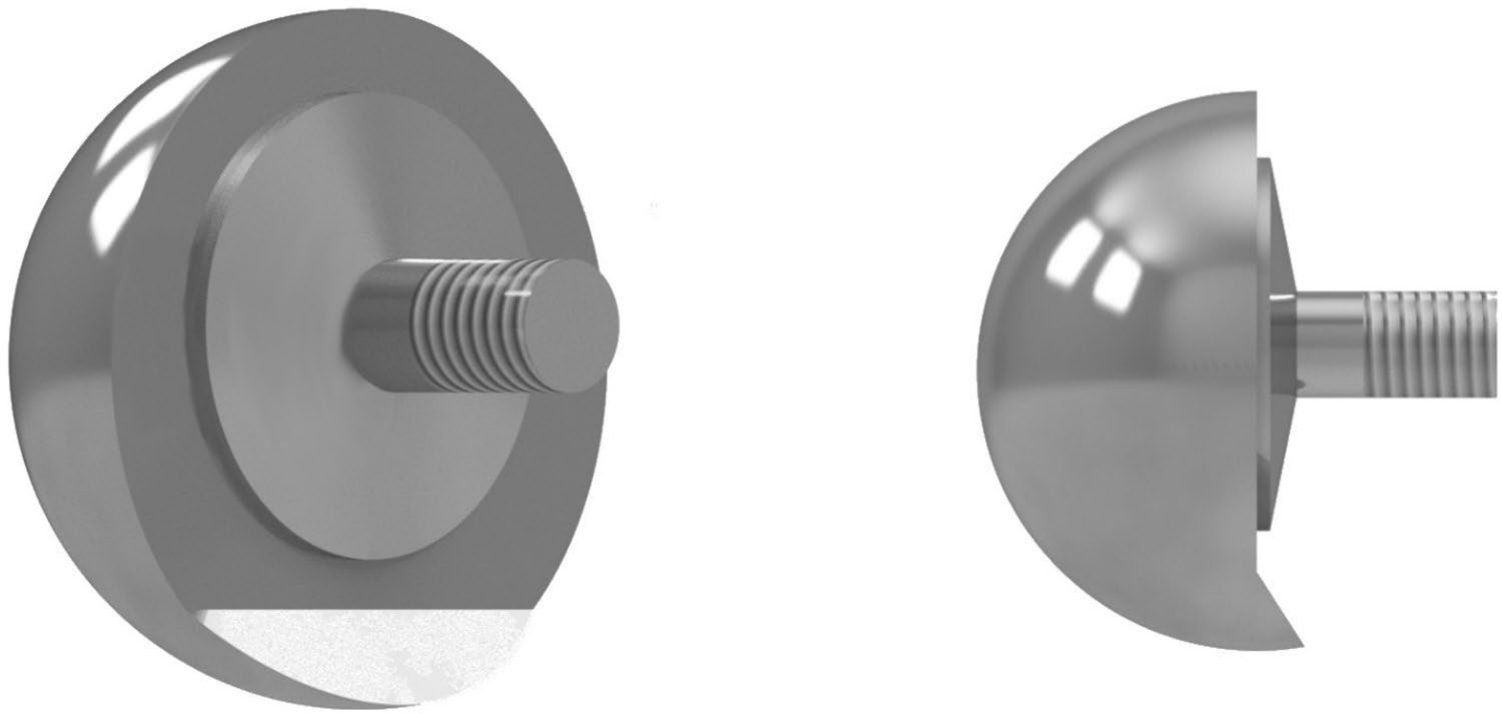
The custom-made eccentric glenosphere with an inferior overhang of 10°

The different implant parameters were glenosphere lateralisation (0 mm vs 6 mm), neck-shaft angle (145° vs 155°), and a custom-made 10° inferiorly inclined glenosphere versus a normal glenosphere without inferior inclination. Since there is currently no available design with built in inclination on the glenosphere in the DePuy Delta Xtend product line, we developed a prototype together with DePuy Synthes which had a 10° inclined overhang at the bottom of the sphere (Fig. 2). For constructional reasons, an eccentric glenosphere with a 42 mm diameter was used as the basis. A 6 mm lateralised glenosphere was achieved with a larger spherical segment thickness. For adaptation of the neck-shaft angle, we used two different modular epiphyses with angles of 145° and 155°, which could be changed on the modular humeral stem. The arm was tested at 30° and 60° of GH abduction in neutral and 30° of internal rotation. Different positions were achieved by the rotation of the arm in force-moment control with target forces of zero and maximum moments of 2 Nm to prevent soft tissue injuries, in accordance with previous studies [23, 25, 26].

The testing order of the eight implant configurations was randomised. In each arm position, the translation stability was evaluated by loading the glenohumeral joint in the anterior direction. An anterior force of 10 N was applied and incrementally increased by 5 N, until the reverse shoulder joint was dislocated. Dislocation was defined as an anterior humeral translation of 21 mm (radius of glenosphere). During testing, the humerus was centred in the glenoid with a 30 N medially oriented force, and the joint was allowed to translate freely in the anterior–posterior, superior–inferior, and medial–lateral directions while all rotations were held constant.

Furthermore, maximum internal rotation was investigated with the arm at 30° and 60° of GH abduction. We defined maximal internal rotation as the angle reached by setting the target value to 2 Nm and a medially oriented centring force of 30 N.

### Radiologic measurements

**Fig. 3:**
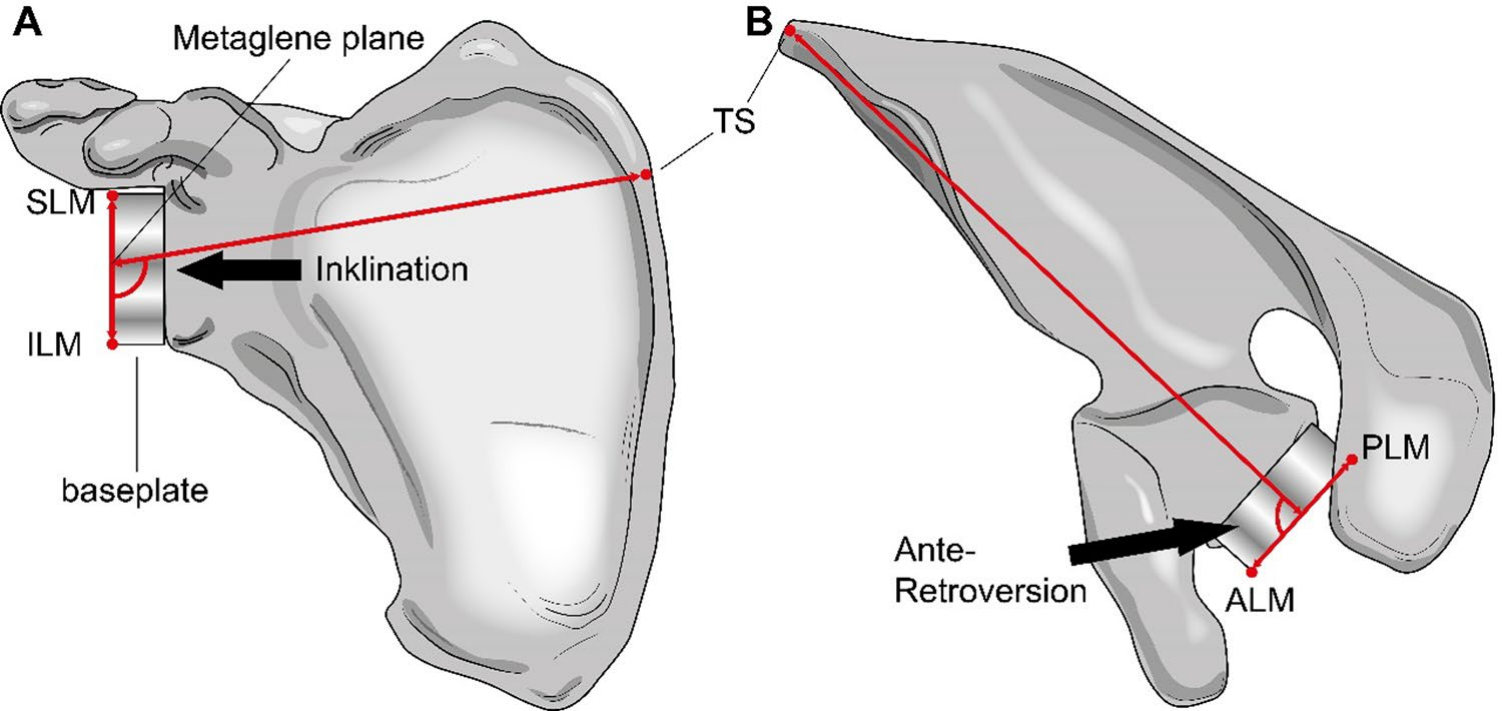
Illustration of the radiological measurements of metaglene inclination (**A**) and ante-retroversion (**B**) angles, respectively. Anatomical landmarks of the scapula are defined in a 3D model of the scapula. Superior landmark metaglene (SLM), inferior landmark metaglene (ILM), trigonum spinae (TS), posterior landmark metaglene (PLM), anterior landmark metaglene (ALM)

Computed tomography (CT) images of the specimens were obtained after the in vitro tests (Revolution HD, General Electric Boston, MA, USA) using a standard protocol. Using special segmentation software (Amira 2020.2, Thermo Fisher Scientific, Waltham, MA, USA), 3D models of each scapula were created. Subsequent to segmentation, 3D bone models were obtained, and 3D measurements were performed using GOM Inspect (GOM GmbH, Braunschweig, Germany). Analogous to the previous study by Pastor et al. [27] the glenocoracoid distance in the anteroposterior (AP) direction and the glenocoracoid distance in the mediolateral (ML) direction were measured. The initial method for measuring these parameters by Dugarte et al. and Van Haver et al. [28, 29] was adapted for application to 3D models. Four landmarks, the superior, inferior, posterior, and anterior aspects of the glenoid rim, were used to create a plane on the glenoid (glenoid plane). The ML distance was measured based on the perpendicular distance, and thus the shortest distance between this plane and the tip of the coracoid. For the AP distance, a second plane was placed perpendicular to the glenoid plane and oriented along the superior-inferior axis of the glenoid plane (SI plane), and the distance between this plane and the coracoid tip was measured. The last two measured parameters were the inclination and ante-retroversion of the metaglene. To this end, an AP axis as well as an SI axis of the metaglene were created using the most anterior, posterior, superior, and inferior landmarks on the metaglene. By means of an axis on the supraspinatus fossa of the scapula and the AP axis of the baseplate, ante-retroversion could be determined as the angle between these two lines. To measure the inclination angle, the SI axis of the metaglene was used instead of the AP axis (Fig. 3).

### Statistical analysis

#### Biomechanical in vitro tests

The biomechanical in vitro test data were statistically evaluated using the programming language R 4.1.0 (R Foundation for Statistical Computing) using the software RStudio 1.4.1717 (RStudio, Inc., Boston, MA, USA). The differences between groups were analysed using 3-way repeated measures analysis of variance. For this purpose, the specimens were classified as repeated measures, and glenosphere lateralisation, neck-shaft angle, glenoid inclination, and arm position were the relevant factors. Tukey’s post hoc test was used to further analyse the significant findings. The significance level was set at *α* = 0.05. The data were presented as means ± standard deviations.

#### CT data/anatomic parameters

Statistical analysis of the CT data was performed using R in RStudio. The previously described anatomical parameters (AP and ML distances, metaglene inclination, and ante-retroversion) were correlated with the dislocation force values of the biomechanical in vitro tests and analysed using a bivariate correlation (Pearson test). Correlations were assessed between the mean anterior dislocation forces and each hardware configuration, arm position, and anatomic parameter. The significance level was set at *α* = 0.05. All data are presented as means ± standard deviations.

## Results

**Fig. 4:**
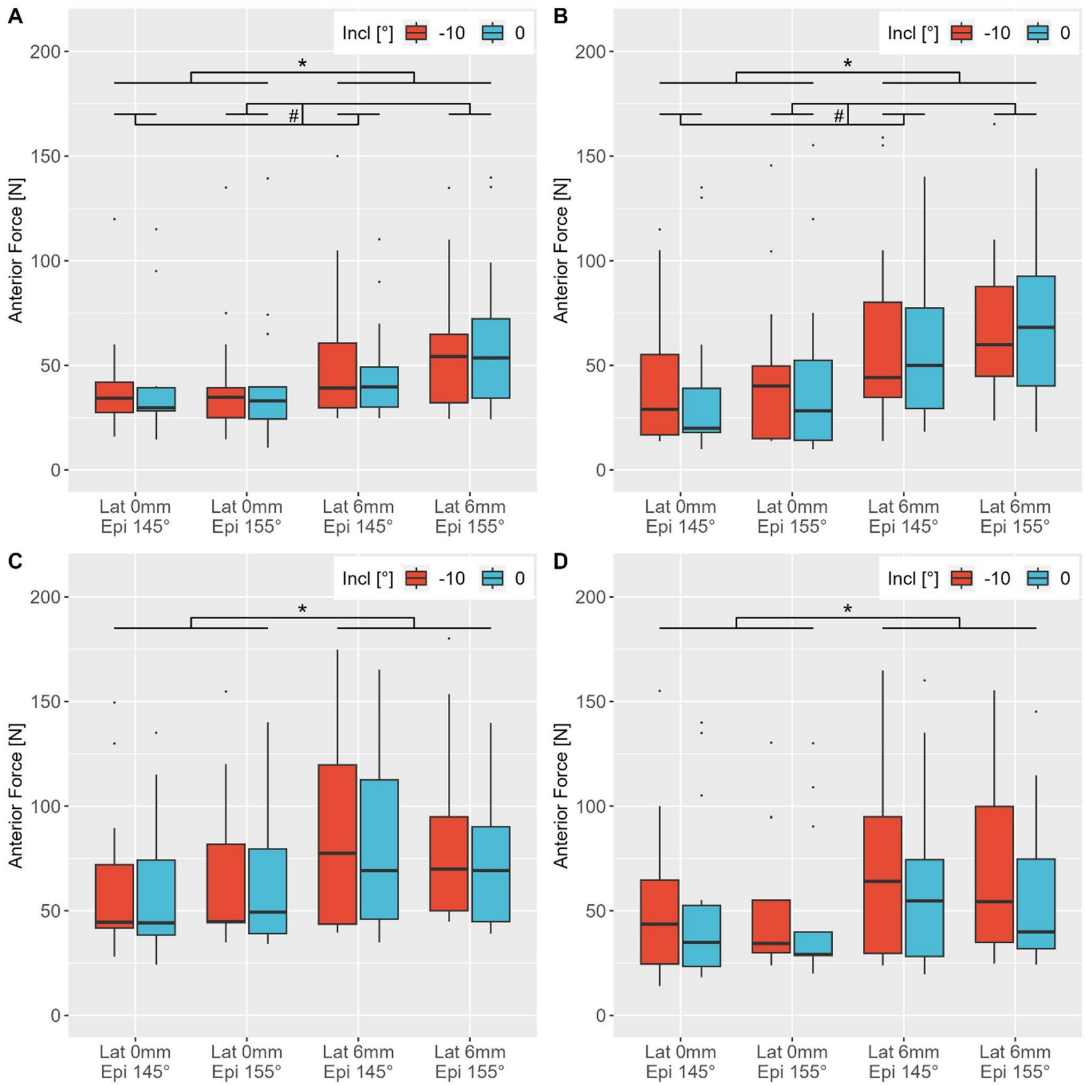
Box plots of the anterior dislocation forces in 30° GH abduction and neutral rotation (**A**), 30° GH abduction and 30° of internal rotation (**B**), 60° GH abduction and neutral rotation (**C**), and 60° GH abduction and 30° of internal rotation (**D**).* indicates significant differences between the configurations with differing glenosphere lateralization (*p* < 0.05). # indicates significant differences between the configurations with differing neck-shaft angles (*p* < 0.05)

In each arm position, lateralisation of the glenosphere by 6 mm had a significant influence on RTSA anterior stability (Fig. 4A–D). Furthermore, in 30° GH abduction with neutral rotation and in 30° GH abduction with 30° internal rotation, the neck-shaft angle showed a significant influence on the anterior stability. In the former arm position, the anterior dislocation force with a neck-shaft angle of 155° was 50.0 ± 33.3 N; and with 145°, 45.0 ± 27.8 N (*p* = 0.012). In the latter arm position, the neck-shaft angle of 155° showed superior stability with 58.1 ± 40.5 N compared with the RTSA with a neck-shaft angle of 145° with 50.6 ± 40.1 N (*p* = 0.002) (Fig. 4A, B). A custom-made and inclined glenospheres did not significantly influence RTSA anterior stability in any arm position. Considering all arm positions and implant configurations together, it was observed that lateralisation of the glenosphere (*p* < 0.001) and arm position (*p* < 0.001) had a significant impact on anterior stability. Particularly, GH abduction of 60° and neutral rotation showed a significant anterior stabilising effect compared with 30° GH abduction with neutral rotation (*p* = 0.0327) and 30° internal rotation (*p* = 0.0358). The 60° abducted arm position required an anterior dislocation force of 71.4 ± 37.7 N. The anterior dislocation force in 30° of GH abduction with neutral rotation was 47.5 ± 30.7 N; and with 30° of internal rotation, 54.33 ± 40.3 N, but this was not significant. With the arm positioned in 30° GH abduction and neutral rotation, lateralisation and the neck-shaft angle together had a significant influence on the anterior stability of the RTSA (*p* = 0.032).

**Fig. 5:**
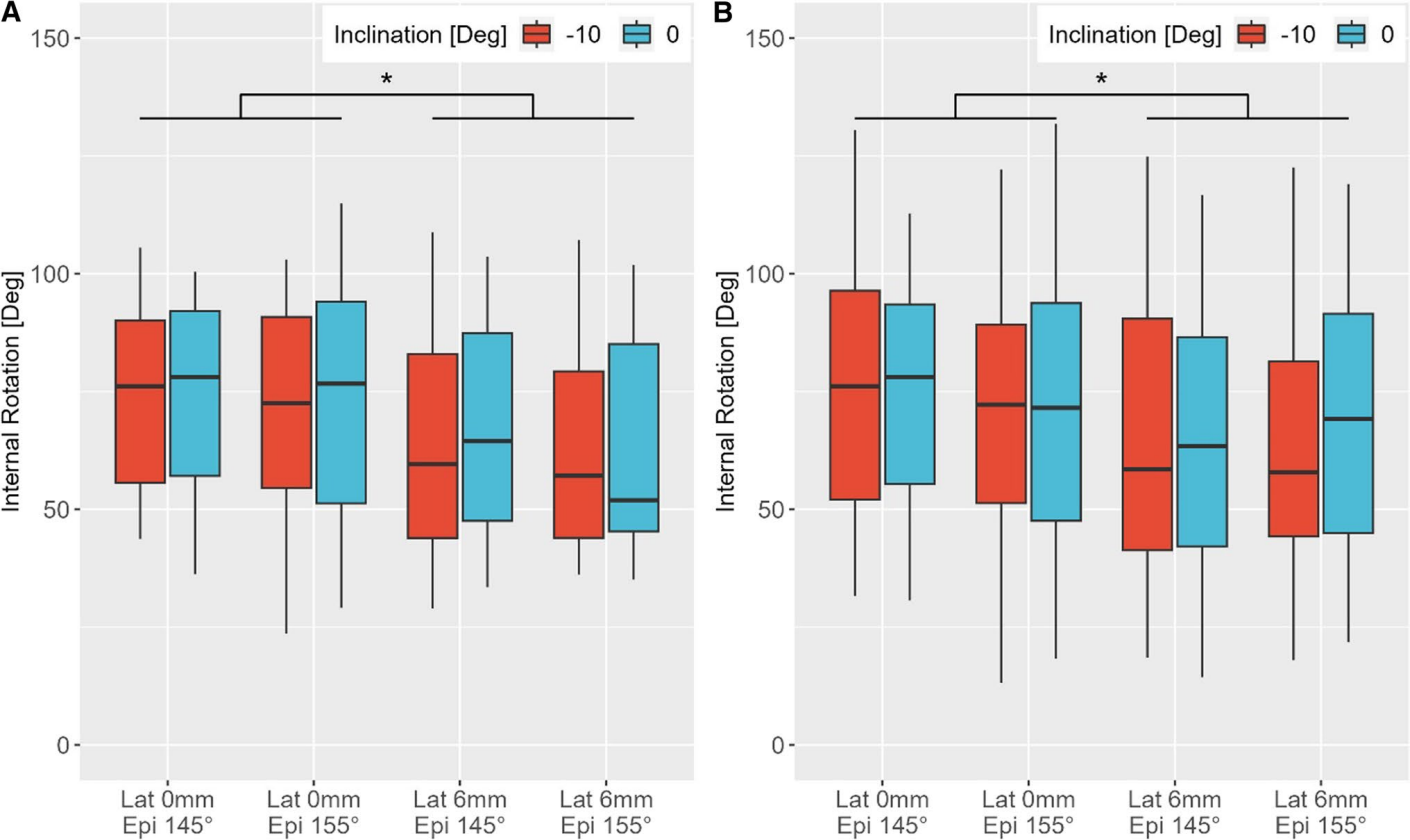
Box plots of the range of motion of internal rotation in 30° GH abduction (**A**) and 60° GH (**B**). * indicates significant differences between the configurations with differing glenosphere lateralization (*p* < 0.05)

The RTSA with glenoid lateralisation of 6 mm led to a significant reduction in internal rotation in 30° and 60° abducted arm positions (Fig. 5).

The CT data showed a mean inferior inclination of the baseplate of 16.1 ± 6° and retroversion of 4.1 ± 6.3°. The mean AP distance was 29.9 ± 3.6 mm; and the ML distance, 14.7 ± 5.1 mm. The correlation analysis between the anterior dislocation forces of the biomechanical testing, and the CT data showed a significant positive correlation between the inclination angles and the anterior dislocation forces for each implant configuration (Table 1). Furthermore, a negative correlation was observed between the AP distance and anterior dislocation forces (Table 1).

**Table 1:** Correlations and *p* values of the glenocoracoid distance in and each hardware configuration and arm position

Hardware configuration				Glenometaglene distance ML (cm)		Glenocoracoid distance AP (cm)		Baseplate inclination (deg)		Baseplate ante-retroversion (deg)		
Arm position	Glenosphere lateralization (mm)	neck-shaft angle (deg)	Glenosphere inclination (deg)	*r* value	*p* value	*r* value	*p* value	*r* value	*p* value	*r* value	*p* value	Dislocation force (N)
00	0	145	− 10	0.1	*p* > 0.05	− 0.4	0.004	0.3	0.018	0.0	*p* > 0.05	49.3
00	0	145	0	0.1	*p* > 0.05	− 0.3	0.012	0.3	0.029	0.0	*p* > 0.05	47.5
00	6	145	− 10	0.1	*p* > 0.05	− 0.4	0.002	0.4	0.005	0.2	*p* > 0.05	66.0
00	6	145	0	0.1	*p* > 0.05	− 0.3	0.009	0.4	0.005	0.2	*p* > 0.05	61.2
00	0	155	− 10	0.0	*p* > 0.05	− 0.3	0.032	0.3	0.011	0.0	*p* > 0.05	51.4
00	0	155	0	0.0	*p* > 0.05	− 0.3	0.040	0.3	0.037	0.0	*p* > 0.05	49.8
00	6	155	− 10	0.1	*p* > 0.05	− 0.5	0.000	0.3	0.034	0.2	*p* > 0.05	69.4
00	6	155	0	0.0	*p* > 0.05	− 0.4	0.003	0.3	0.013	0.1	*p* > 0.05	66.8
30	0	145	− 10	0.1	*p* > 0.05	− 0.4	*p* > 0.05	0.2	*p* > 0.05	0.0	*p* > 0.05	40.9
33	0	145	− 10	− 0.2	*p* > 0.05	− 0.4	*p* > 0.05	0.0	*p* > 0.05	0.2	*p* > 0.05	41.1
60	0	145	− 10	0.3	*p* > 0.05	− 0.3	*p* > 0.05	0.6	0.032	0.0	*p* > 0.05	63.0
63	0	145	− 10	0.4	*p* > 0.05	− 0.5	*p* > 0.05	0.4	*p* > 0.05	0.0	*p* > 0.05	52.2
30	0	145	0	− 0.1	*p* > 0.05	− 0.3	*p* > 0.05	0.2	*p* > 0.05	0.0	*p* > 0.05	40.3
33	0	145	0	− 0.2	*p* > 0.05	− 0.3	*p* > 0.05	0.1	*p* > 0.05	0.1	*p* > 0.05	39.5
60	0	145	0	0.2	*p* > 0.05	− 0.3	*p* > 0.05	0.5	0.044	− 0.1	*p* > 0.05	59.8
63	0	145	0	0.3	*p* > 0.05	− 0.4	*p* > 0.05	0.4	*p* > 0.05	0.0	*p* > 0.05	50.6
30	6	145	− 10	0.0	*p* > 0.05	− 0.4	*p* > 0.05	0.3	*p* > 0.05	0.0	*p* > 0.05	51.5
33	6	145	− 10	− 0.1	*p* > 0.05	− 0.5	*p* > 0.05	0.1	*p* > 0.05	0.3	*p* > 0.05	63.0
60	6	145	− 10	0.3	*p* > 0.05	− 0.3	*p* > 0.05	0.5	*p* > 0.05	0.2	*p* > 0.05	83.2
63	6	145	− 10	0.3	*p* > 0.05	− 0.5	*p* > 0.05	0.6	0.023	0.3	*p* > 0.05	67.7
30	6	145	0	0.0	*p* > 0.05	− 0.4	*p* > 0.05	0.4	*p* > 0.05	0.0	*p* > 0.05	47.2
33	6	145	0	− 0.2	*p* > 0.05	− 0.3	*p* > 0.05	0.1	*p* > 0.05	0.3	*p* > 0.05	58.8
60	6	145	0	0.2	*p* > 0.05	− 0.2	*p* > 0.05	0.5	*p* > 0.05	0.2	*p* > 0.05	79.5
63	6	145	0	0.4	*p* > 0.05	− 0.5	*p* > 0.05	0.6	0.031	0.3	*p* > 0.05	60.6
30	0	155	− 10	0.0	*p* > 0.05	− 0.3	*p* > 0.05	0.3	*p* > 0.05	0.0	*p* > 0.05	41.6
33	0	155	− 10	− 0.3	*p* > 0.05	− 0.4	*p* > 0.05	0.0	*p* > 0.05	0.0	*p* > 0.05	45.2
60	0	155	− 10	0.2	*p* > 0.05	− 0.2	*p* > 0.05	0.5	0.038	0.0	*p* > 0.05	66.8
63	0	155	− 10	0.3	*p* > 0.05	− 0.3	*p* > 0.05	0.6	0.042	0.0	*p* > 0.05	52.1
30	0	155	0	0.0	*p* > 0.05	− 0.3	*p* > 0.05	0.2	*p* > 0.05	− 0.1	*p* > 0.05	41.1
33	0	155	0	− 0.2	*p* > 0.05	− 0.3	*p* > 0.05	0.0	*p* > 0.05	0.1	*p* > 0.05	44.4
60	0	155	0	0.2	*p* > 0.05	− 0.2	*p* > 0.05	0.4	*p* > 0.05	− 0.1	*p* > 0.05	65.1
63	0	155	0	0.3	*p* > 0.05	− 0.3	*p* > 0.05	0.5	*p* > 0.05	0.0	*p* > 0.05	48.2
30	6	155	− 10	0.1	*p* > 0.05	− 0.5	*p* > 0.05	0.4	*p* > 0.05	0.1	*p* > 0.05	56.4
33	6	155	− 10	− 0.2	*p* > 0.05	− 0.5	*p* > 0.05	0.0	*p* > 0.05	0.1	*p* > 0.05	71.2
60	6	155	− 10	0.4	*p* > 0.05	− 0.4	*p* > 0.05	0.4	*p* > 0.05	0.3	*p* > 0.05	82.8
63	6	155	− 10	0.3	*p* > 0.05	− 0.6	0.035	0.5	*p* > 0.05	0.3	*p* > 0.05	68.8
30	6	155	0	0.1	*p* > 0.05	− 0.5	*p* > 0.05	0.5	*p* > 0.05	0.0	*p* > 0.05	60.9
33	6	155	0	− 0.1	*p* > 0.05	− 0.5	*p* > 0.05	0.2	*p* > 0.05	0.3	*p* > 0.05	71.5
60	6	155	0	0.0	*p* > 0.05	− 0.2	*p* > 0.05	0.2	*p* > 0.05	0.1	*p* > 0.05	74.1
63	6	155	0	0.0	*p* > 0.05	− 0.4	*p* > 0.05	0.5	*p* > 0.05	0.2	*p* > 0.05	60.5

00: independent of arm position / averaged over all arm positions

30: 30° GH abduction and neutral internal rotation

33: 30° GH abduction and 30° internal rotation

60: 60° GH abduction and 0° internal rotation

63: 60° GH abduction and 30° internal rotation

AP, anteroposterior; ML, mediolateral

## Discussion

This study aimed to investigate the influence of a custom-made inclined glenosphere on RTSA anterior stability. The results of this study did not support our hypothesis because the custom-made inclined glenosphere did not lead to superior anterior stability. However, the inferior inclination of the baseplate was positively correlated with anterior stability.

The impetus for our study on this controversial topic was the two studies by Randelli et al. and Tashjian et al. [13, 14]. Randelli et al. [13], who performed a retrospective study of 33 patients, calculated the glenoid and glenosphere inclinations on anteroposterior and axillary radiographs. Three dislocations were observed in the collective. Stable shoulders had a mean inferior inclination of 10.2°; consequently, the authors concluded that this inclination presented a reduced risk of dislocation when compared with neutral tilt. A further study by Tashjian et al. [14] reported instability in the 13 shoulders of 97 patients. The factors associated with instability were greater superior baseplate inclination and greater change towards superior inclination from pre-operative to post-operative. The findings of the two studies could be partially supported by our results because the observed inferior inclination of the baseplate showed a positive correlation with RTSA anterior stability. However, the glenosphere inclined at 10° did not improve stability. The reason for this discrepancy could be that the biomechanical testing was performed in 30° and 60° GH abduction and not in the neutral position or adduction. In a previous biomechanical study, some of the testing arm positions were not achieved because of high soft tissue tension [12, 24, 25, 30]. With our biomechanical testing protocol, we were able to obtain reliable and reproducible results. To test the shoulder in neutral position or adduction, the soft tissue release and resection would have had to be significantly expanded to a higher level than would usually be performed during surgery. Further, Randelli et al. and Tashjian et al. [13, 14] performed the implantation of RTSA with medialized centre of rotation (COR) with medialized glenoid and medialized humerus (MGMH). This configuration was associated with greater rotator cuff muscle shortening and less deltoid wrapping and could increase the risk of dislocation [31]. Perhaps the combination of the medialized COR, superior tilt of the baseplate as well as subscapularis tenotomy caused the increased risk of dislocation in these studies of Randelli et al. and Tashjian et al. In this study, different configurations with different COR were used, but no significant differences were found with MGMH with normal or inclined glenosphere. In case of lateralized glenoid and lateralized humerus (LGLH) or lateralized glenoid and medialized humerus (LGMH), the lateralisation could increase the anterior stability so that the inclined glenosphere only played a subordinate role. However, in the case–control study by Bechtold et al., [15] which included 34 patients with a dislocation after RTSA and 102 patients as the control group, no increased risk related to glenoid inclination was observed. No differences were found between the stable and unstable shoulders in either the glenoid inclination angle or the change in inclination angle from preoperative to postoperative. Furthermore, this study did not show an increased risk of dislocation in shoulders with a superior glenoid inclination [15]. The inferior tilt of the lateralised or standard glenosphere minimises the forces at the baseplate bone interface, which potentially can cause implant loosening [16], but the opposite was observed for the eccentric inferior tilted glenospheres in the computer model used in this study. These findings could minimise the mechanical failure of the baseplate [16]. In addition to the advantage of possible improvements in stability, an inferior inclination also has disadvantages. Owing to the inferior tilt of the baseplate, the distance to the scapula neck was shortened, which increased the risk of impingement [17]. Clinically, this complication can lead to impingement-associated instability and onlay erosion. Furthermore, Tashjian et al. [32] observed in a biomechanical study that an inferiorly tilted glenosphere increased deltoid abduction forces.

Glenoid lateralisation has been associated with improved range of motion in biomechanical and 3D model studies [32–35]. Clinical studies have also shown that glenoid lateralisation improves range of motion, especially external rotation [36–38]. Werner et al. observed improved internal rotation in patients with 6 and 8 mm lateralisation compared with patients with lateralisation of less than 6 mm [38]. In contrast, in our study, decreased internal rotation was found in the case of 6 mm glenoid lateralisation. This could be explained by the increased tension of the infraspinatus and teres minor tendon caused by glenosphere lateralisation limiting internal rotation.

The anterior dislocation forces of the RTSA with a custom-made inclined glenosphere were also negatively correlated with AP distance to the tip of the coracoid. This is in agreement with the results of a previous study [27]. This study also confirmed the findings of a previous investigation [27], which found that glenoid lateralisation had a significant influence on RTSA anterior stability in all arm positions. In particular at 30° abduction and neutral rotation, the neck-shaft-angle and the lateralization together had a significant influence on anterior dislocation force. Because this effect was only observed in this one arm combination, the transfer to the clinic is only very limited. It is known that neck-shaft angle of 135° did not led to a higher dislocations rate in clinical investigations. Furthermore, a humeral lateralization is preferred clinically to decrease the risk of scapular notching and dislocation [39].

However, this study has limitations. First, it was a biomechanical in vitro study with human shoulder specimens at time point zero; therefore, potential biological effects affecting the anterior stability of the RTSA, such as soft tissue healing, prosthetic ingrowth, and muscle tension, could not be investigated. Second, biomechanical testing was only performed with one type of RTSA; therefore, it was not possible to make any statements about other prostheses, especially implants with a humeral onlay design. Third, biomechanical testing was not performed in neutral GH abduction or adduction, so the potential maximum benefit of the inclined glenosphere could not be determined. To investigate neutral GH abduction or adduction, biomechanical testing and specimen preparation must be adapted, and further studies are needed. Furthermore, the focus was only on the glenohumeral joint, as we could not investigate the influence of the scapula movement due to the fixed scapula in our test setup. In the end, it must also be noted that we only examined passive joint stability, thus neglecting the influence of actively engaged muscles of the rotator cuff or deltoid on joint stability. In conclusion, the study did not prove that the custom-made 10° inclined glenosphere had a significant influence on RTSA anterior stability, but glenoid inferior inclination showed a significant positive correlation with the anterior dislocation force, indicating that inclination has an influence on anterior stability. Furthermore, the study observed that internal rotation was reduced in the case of an RTSA with 6 mm glenoid lateralisation.
